# Decreasing Hydrogen Content within Zirconium Using Au and Pd Nanoparticles as Sacrificial Agents under Pressurized Water at High Temperature

**DOI:** 10.3390/ma16186164

**Published:** 2023-09-11

**Authors:** Yeon Ju Lee, Juhee Ha, Su Ji Choi, Hyeok Il Kim, Sumin Ryu, Youngsoo Kim, Young-Sang Youn

**Affiliations:** Department of Chemistry, Yeungnam University, Daehak-ro 280, Gyeongsan 38541, Gyeongbuk, Republic of Korea; lyj78688@yu.ac.kr (Y.J.L.); wngml9571@yu.ac.kr (J.H.); ctw1217@ynu.ac.kr (S.J.C.); tbq123@yu.ac.kr (H.I.K.); ryusm330@yu.ac.kr (S.R.)

**Keywords:** zirconium, hydride, nanoparticle, dry storage, simulated pressurized water reactor conditions

## Abstract

Decreasing hydride-induced embrittlement of zirconium-based cladding is a significant challenge for the successful dry storage of spent nuclear fuel. Herein, to radically minimize hydride-induced embrittlement, we used nanoparticles as sacrificial agents with a greater affinity than zirconium for hydrogen. Corrosion experiments in the presence of gold (Au) and palladium (Pd) nanoparticles under simulated pressurized water reactor (PWR) conditions revealed that the hydrogen content of the zirconium samples was remarkably reduced, with a maximum decrease efficiency of 53.9% using 65 nm Au and 53.8% using 50 nm Pd nanoparticles. This approach provides an effective strategy for preventing hydride-induced embrittlement of zirconium-based cladding.

## 1. Introduction

Zirconium-based alloys consisting of 95% or more zirconium are typically used as nuclear fuel cladding materials in pressurized water reactors (PWRs) owing to their superior mechanical properties, corrosion resistance, chemical stability, heat transfer, and thermal absorption neutron cross-section compared to other alloys [1,2,3,4,5,6,7,8]. During reactor operation, the cladding is constantly in contact with the primary circuit water to transfer the thermal energy produced by the nuclear fuel to the water for the generation of electrical energy [9]. The corrosion reaction with the primary water produces hydrogen [10,11,12,13,14,15], which accumulates in the cladding owing to the high hydrogen affinity of zirconium [16]. Consequently, hydrogen ingress into the cladding is inevitable during reactor service [17,18,19]. When the hydrogen concentration exceeds the terminal solid solubility (TSS) limit for precipitation, the hydrogen absorbed in the cladding agglomerates as hydrides in the zirconium matrix [5,12,20,21]. These hydrides preferentially orient along the circumferential direction rather than the radial direction in cylindrical cladding during normal operation [5,11]. Although hydrides embedded along the circumferential orientation reduce the cladding ductility, the cladding can endure deformation by the circumferential hydrides [5,22]. However, when the spent nuclear fuel is withdrawn from the spent fuel pool for dry storage after a certain period, the hydrides reorient from the circumferential direction to the radial direction because the applied tensile hoop stress on the cladding exceeds the threshold stress under dry storage conditions [11,23,24,25,26]. Radial hydrides severely embrittle cladding, not only deteriorating the mechanical properties such as ductility and fracture toughness [5,10,11,22,27,28,29], but also increasing the ductile–brittle transition temperature (DBTT) [22,29]. Therefore, preventing the reorientation of hydrides into the radial direction in the cladding is crucial for maintaining cladding integrity during the dry storage of spent nuclear fuel [12,19,26,30,31].

One way to reduce radial hydride formation in zirconium-based cladding under PWR conditions is to utilize sacrificial materials capable of capturing hydrogen near the cladding. Several studies have examined the efficacy of metal nanoparticles, including Au, Pd, and Pd-containing alloy nanoparticles, for hydrogen capture or dissociation in non-nuclear-related applications [32,33,34,35]. Although Au nanoparticles do not directly absorb hydrogen molecules, the strong catalytic activity on the nanoparticle surface contributes to the dissociation of the adsorbed hydrogen molecules into atomic hydrogen, which exhibits a strong affinity for the Au surface [35,36,37]. In contrast, Pd and Pd-containing alloy nanoparticles are known for their ability to absorb and convert hydrogen into a Pd-hydride form [33,34,38]. Single crystal α-phase Pd nanoparticles can absorb hydrogen under moderate temperature and pressure conditions. As the amount of hydrogen absorbed by the Pd nanoparticles increases, the lattice distance between the Pd atoms expands, resulting in the formation of β-phase Pd nanoparticles known as Pd-hydrides [33,34,39]. Therefore, both Au and Pd nanoparticles are promising materials for capturing hydrogen, albeit via different mechanisms.

**Figure 1:**
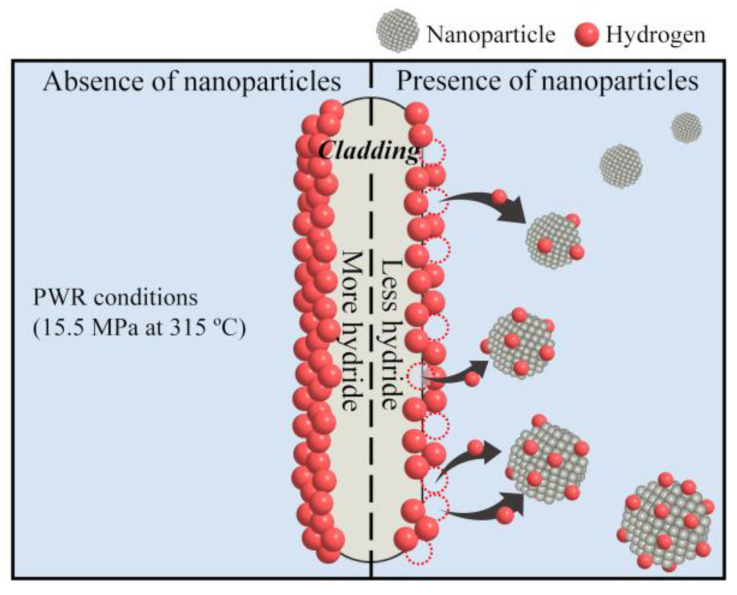
Schematic of the influence of metal nanoparticles on hydride formation on zirconium-based cladding under PWR conditions.

This study is the first to utilize Au and Pd nanoparticles as sacrificial agents to preferentially capture hydrogen and reduce the hydrogen absorption of zirconium-based cladding under simulated PWR conditions, which consequently minimizes the hydride-induced embrittlement of the cladding. The core concept of the proposed strategy is schematically illustrated in Figure 1. In the absence of nanoparticles, the zirconium-based cladding is highly vulnerable to corrosion owing to the high absorption of hydrogen on its surface and throughout its bulk under PWR conditions. However, in the presence of nanoparticles, the hydrogen produced by the reaction between the cladding and water is distributed between the cladding and nanoparticles under PWR conditions, thereby decreasing hydride formation on the cladding. The hydrogen content of the zirconium after corrosion experiments using Au or Pd nanoparticles under simulated PWR conditions was significantly reduced compared to that without nanoparticles, indicating that the hydrogen produced on the zirconium surface was efficiently captured by the Au and Pd nanoparticles dispersed in the water. These findings suggest that hydrogen is preferentially absorbed on the Au and Pd nanoparticles over the zirconium. Therefore, a significant reduction in radial-hydride-driven embrittlement due to hydride reorientation in the cladding during the dry storage of spent nuclear fuel can potentially be achieved by dispersing Au and Pd nanoparticles in the coolant.

## 2. Materials and Methods

Zirconium pellets with a diameter of 10 mm and a thickness of 1 mm were prepared by mechanically pressing zirconium powder (99.5% purity, Se-Jong Materials Co., Ltd., Incheon, Republic of Korea) at 20.7 MPa. The fabricated zirconium pellet was heated at 850°C for a week under an argon (99.999% purity) atmosphere in a quartz tube furnace (LF-GT530, LK Lab Korea, Namyangju-si, Republic of Korea). After heating, the zirconium pellets were polished with 3000 grit SiC paper, resulting in a mean density of 4519 kg/m^3^. The hydrogen content of the as-prepared zirconium pellet was 13 μg/g, which was similar to the values reported in the literature for zirconium-based cladding [2,18].

The Au and Pd nanoparticles were prepared via colloidal synthesis. Two different synthesis methods were used to prepare the spherical Au nanoparticles. Small spherical Au nanoparticles (13 and 25 nm) were synthesized using the Frens method by adjusting the HAuCl_4_/citrate/AgNO_3_ ratio to an appropriate value [40]. Large Au nanoparticles (65 and 109 nm) were prepared using the seed-growth method [41,42]. For the synthesis of Pd nanoparticles, the nucleation and growth method was used to synthesize 12 nm Pd nanoparticles [43], whereas the ethanol reduction method was used to synthesize 50 and 108 nm Pd nanoparticles [44]. The synthetic procedures for all the metal nanoparticles are described in detail in the Supplementary Materials.

**Figure 2:**
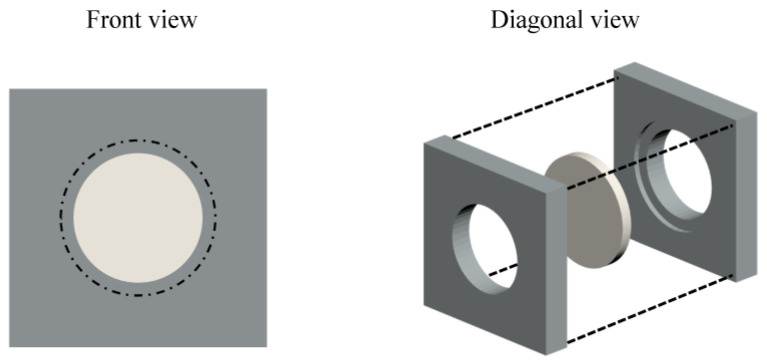
Schematic of the front and diagonal views of a zirconium pellet mounted on the sample holder for corrosion experiments; 8 mm diameter zones on both sides of the pellet were in contact with water during the corrosion test.

The corrosion tests of the zirconium pellets were conducted in 350 mL of deionized water (New P.NIX Power water purification system, Daihan Scientific, Wonju, Republic of Korea) with/without nanoparticles. The pressure and temperature were maintained at 15.5 ± 0.5 MPa and 315°C, respectively, for 24 h under continuous stirring at 200 rpm in a 700 mL stirred autoclave system (Ilshin Autoclave, Daejeon, Republic of Korea). An appropriate amount of nitrogen gas (99.999% purity) was injected into the system before the temperature was increased to attain the target pressure at 315°C. The scheme in Figure 2 shows a zirconium pellet mounted on a sample holder for the corrosion experiments. An 8 mm diameter area on both sides of the zirconium pellet was exposed to the deionized water during the corrosion tests.

X-ray diffraction (XRD) data were obtained in the range of 20–80° with a scanning step of 0.02° for 0.6 s using a MiniFlex600 system (Rigaku, Japan) with Cu K_α_ radiation operated at 15 mA and 40 kV. Raman spectra were acquired over the range of 130–800 cm^−1^ using an NS240 Raman spectrometer (Nanoscope Systems, Daejeon, Republic of Korea) with a continuous-wave diode-pumped laser at a wavelength of 532 nm and an exposure time of 30 s. Transmission electron microscopy (TEM) images were acquired using an H-7600 system (Hitachi, Tokyo, Japan) at an accelerating voltage of 120 kV. The optical properties of the metal nanoparticles were characterized using a UV-1800 spectrophotometer (Shimadzu, Kyoto, Japan) in a scan range of 300–800 nm. The hydrogen content within the zirconium pellet was determined using a hydrogen analyzer (RH-404, LECO Corporation, St. Joseph, MI, USA) with a sensitivity of ±5 μg/g [26].

## 3. Results

The XRD pattern of a zirconium pellet before the corrosion experiment is shown in Figure S1. At room temperature and atmospheric pressure, zirconium exhibits a hexagonal crystal structure [2,6], and the diffraction patterns associated with hexagonal Zr can be clearly observed in Figure S1.

**Figure 3:**
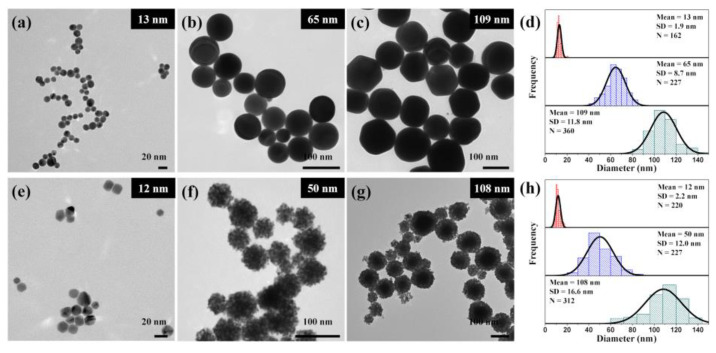
Representative TEM images of (**a**–**c**) Au and (**e**–**g**) Pd nanoparticles. Histograms of the (**d**) Au and (**h**) Pd nanoparticle size distribution; SD and N denote the standard deviation and number of particles counted, respectively.

For the corrosion test of the zirconium pellets, the size of the Au and Pd nanoparticles was varied. Au nanoparticles with four different diameters and Pd nanoparticles with three different diameters were examined. Figure 3 shows the TEM images of the various synthesized Au and Pd nanoparticles. The nanoparticle diameters were determined by randomly selecting nanoparticles from different regions of the TEM grid. The mean diameters of the Au nanoparticles (Figure 3a–c and Figure S2) were 13 ± 1.9 nm, 25 ± 3.1 nm, 65 ± 8.7 nm, and 109 ± 11.8 nm, respectively, and those of the Pd nanoparticles (Figure 3e–g) were 12 ± 2.2 nm, 50 ± 12.0 nm, and 108 ± 16.6 nm, respectively.

**Figure 4:**
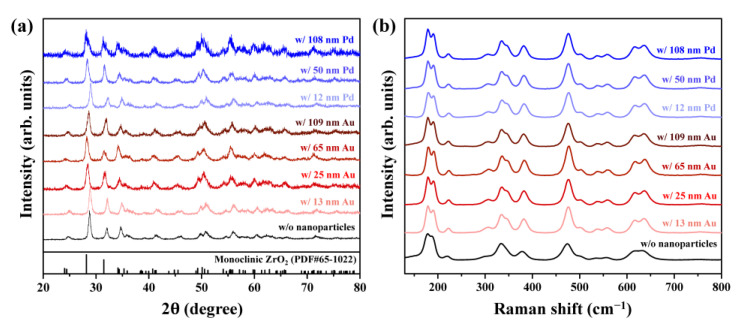
(**a**) XRD and (**b**) Raman data of the zirconium pellets after the corrosion tests. Bottom panel in (**a**): Diffraction pattern of monoclinic ZrO_2_ (PDF#65-1022) obtained from the PDF-2 database.

Figure 4 shows the XRD and Raman data of the zirconium specimens after the corrosion experiments with/without various sizes of synthesized Au and Pd nanoparticles. Diffraction peaks corresponding to a monoclinic ZrO_2_ crystal structure were observed in all pellets, regardless of the nanoparticles used (Figure 4a) [45,46]. This monoclinic ZrO_2_ crystal structure was also confirmed by the Raman profiles (Figure 4b) [45,47]. Zirconium is known to absorb hydrogen while being oxidized to ZrO_2_ by the corrosion reaction with the primary water during reactor operation [21,26,48]. Therefore, the ZrO_2_ observed in all the pellets arose from the reaction of zirconium with water under PWR conditions.

**Figure 5:**
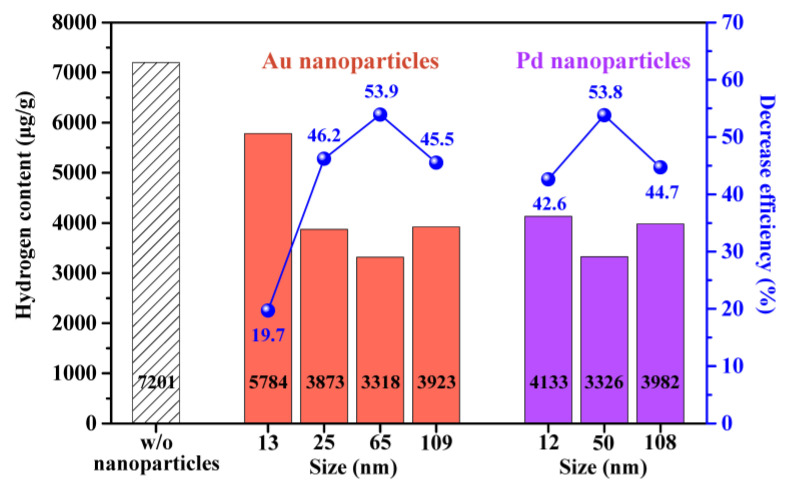
Hydrogen content within the zirconium pellets and the corresponding decrease efficiency after the corrosion experiments with/without various sizes of the synthesized Au and Pd nanoparticles.

The hydrogen content of the zirconium pellets was determined after the corrosion tests with/without various sizes of synthesized Au and Pd nanoparticles (Figure 5). In the absence of nanoparticles, the hydrogen content of the zirconium after the corrosion test was 7201 μg/g, which was significantly higher than the 13 μg/g value observed before the corrosion test. This indicated that a significant amount of the hydrogen produced under the simulated PWR conditions had accumulated in the zirconium. In the presence of nanoparticles, the hydrogen content of the zirconium samples was remarkably reduced by an average of 4048 μg/g, an average decrease efficiency of 43.8%, regardless of the type and size of the nanoparticles, owing to the excellent hydrogen absorption abilities of the Au and Pd nanoparticles [32,33,34,35,36,37,38]. The hydrogen capture ability of nanoparticles typically increases with decreasing nanoparticle size, regardless of the type of nanoparticle [35,49,50,51]. However, in this study, the midsized Au and Pd nanoparticles resulted in a greater decrease in the hydrogen content of the zirconium specimens, with values of 3883 μg/g using the 65 nm Au and 3875 μg/g using the 50 nm Pd nanoparticles (a decrease efficiency of 53.9 and 53.8%, respectively), than that observed for the 13 nm Au, 25 nm Au, and 12 nm Pd nanoparticles. In addition, when larger 109 nm Au and 108 nm Pd nanoparticles were used, the effect of the nanoparticles on the reduction in the hydrogen concentration within the zirconium pellets declined. In general, metal nanoparticles primarily capture hydrogen through surface sorption, and the nanoparticle’s surface plays a crucial role in this process. Notably, all the nanoparticles used in our experiments were exposed to high temperature and pressure. During corrosion tests conducted under PWR conditions, nanoparticles can potentially destabilize, rendering them susceptible to destruction or agglomeration. Consequently, the actual size of the metal nanoparticles during the reaction under PWR conditions may differ from the original size determined from the TEM images. Smaller nanoparticles in the range of 12–25 nm can succumb to the prolonged high-temperature and -pressure conditions encountered in PWR systems. In contrast, the midsized nanoparticles used in our experiments (65 nm for Au and 50 nm for Pd) maintained their particulate structure under the PWR conditions for a longer period than the smaller particles, which enabled them to capture more hydrogen. In addition, particles larger than 100 nm exhibited a slightly lower hydrogen capture capacity owing to the size effect. As a result, nanoparticles larger than the threshold size exhibited the same trend as those reported in the literature, namely the hydrogen capture ability increased as the particle size decreased [35,49,50,51]. Combining our experimental findings with those in the literature, we concluded that the optimal hydrogen capture performance is achieved using 65 nm Au or 50 nm Pd nanoparticles under the simulated PWR conditions. This observation aligns with trends previously reported in the literature and can explain the volcano-like trend in the decrease efficiency (Figure 5).

In addition, we monitored the UV–vis absorption spectra of the nanoparticle-containing water before and after the reaction to compare their optical properties (Figure S3). The absorption spectra of the recovered water after the reaction exhibited no noticeable peaks for any of the nanoparticle sizes. Instead, small and broad absorption features were observed across the entire wavelength range. When Au and Pd nanoparticles undergo significant aggregation, their behavior resembles that of bulk materials. Consequently, the nanoparticles lose their unique nanoscale optical properties, resulting in the disappearance of absorption features in the visible-light region. The data in Figure 5 and Figure S3 suggest that the nanoparticles were unable to maintain an intact structural framework under the PWR conditions and were prone to significant aggregation. Consequently, we postulated that the process by which hydrogen was captured on the nanoparticles was highly active during the initial phase of the reaction. However, as the reaction time increases, the hydrogen capture ability of the nanoparticles may degrade owing to structural deformation.

## 4. Conclusions

We investigated the decrease in hydrogen content within zirconium using Au and Pd nanoparticles as sacrificial agents under simulated PWR conditions. The composition of all the zirconium pellets was changed from hexagonal Zr metal to monoclinic ZrO_2_ after the corrosion experiments owing to their reaction with water in which the zirconium absorbed hydrogen while being oxidized. After the corrosion experiments in the presence of Au or Pd nanoparticles, the hydrogen content of the zirconium samples decreased significantly. In particular, the 65 nm Au and 50 nm Pd nanoparticles resulted in the lowest hydrogen contents among the zirconium samples, with contents of 3318 and 3326 μg/g and a corresponding decrease efficiency of 53.9 and 53.8%, respectively. Our discovery of the successful application of Au and Pd nanoparticles as sacrificial agents for preferential entrapment of hydrogen over zirconium is unprecedented in the field of nuclear materials and can be utilized as a strategy to significantly minimize the hydride-induced embrittlement of zirconium-based cladding.

## Data Availability

Not applicable.

## Supplementary Materials

The following supporting information can be downloaded at: https://www.mdpi.com/article/10.3390/ma16186164/s1, Detailed synthetic procedures; Figure S1: XRD spectrum of a zirconium pellet obtained before the corrosion test.; Figure S2: Representative TEM images of Au and Pd nanoparticles.; Figure S3: UV–vis absorption spectra for 109 nm Au and 108 nm Pd nanoparticles before and after the corrosion test.
