# Supplementary material for: Decreasing Hydrogen Content within Zirconium Using Au and Pd Nanoparticles as Sacrificial Agents under Pressurized Water at High Temperature
Source: Materials (Basel). 2023 Sep 11;16(18):6164. doi: 10.3390/ma16186164 (PMC10532928; doi:10.3390/ma16186164)
Supplement: Supplementary file 1 [file materials-16-06164-s001.zip › materials-2578525-supplementary.pdf]

Supplementary Information

Decreasing Hydrogen Content within Zirconium Using Au and Pd Nanoparticles as Sacrificial Agents under Pressurized Water at High Temperature

Yeon Ju Lee †, Juhee Ha †, Su Ji Choi, Hyeok Il Kim, Sumin Ryu, Youngsoo Kim * and Young-Sang Youn *

Department of Chemistry, Yeungnam University, Daehak-ro 280, Gyeongsan 38541, Gyeongbuk, Republic of Korea; lyj78688@yu.ac.kr (Y.J.L.); wngml9571@yu.ac.kr (J.H.); ctw1217@ynu.ac.kr (S.J.C.); tbq123@yu.ac.kr (H.I.K.); ryusm330@yu.ac.kr (S.R.)

* Correspondence: kimys6553@yu.ac.kr (Y.K.); ysyoun@yu.ac.kr (Y.-S.Y.); Tel.: +82-53-810-2355 (Y.K.); Tel.: +82-53-810-2357 (Y.-S.Y.)

† These authors contributed equally to this work.

Detailed synthetic procedures

Materials

Au (III) chloride trihydrate ($HAuCl_4 \cdot 3H_2O$, ≥49% metal basis), sodium borohydride ($NaBH_4$, 98%), ascorbic acid (AA, 99%), palladium (II) sodium chloride (Na_2PdCl_4, 99.99% trace metal basis) were purchased from Sigma-Aldrich (USA). Cetyl trimethyl ammonium bromide (CTAB, 98%), cetyl trimethyl ammonium chloride (CTAC, 98%), palladium (II) acetate ($Pd(OAc)_2$, 98%), dimethyl sulfoxide (DMSO, 99%) were obtained from Tokyo Chemical Industry (TCI, Japan). Trisodium citrate dihydrate (99% purity) was purchased from Alfa Aesar (USA). Silver nitrate ($AgNO_3$, 99.8%), polyvinylpyrrolidone (PVP, K30), ethylene glycol (99%) were purchased from DaeJung Chemicals (Republic of Korea). All chemicals used were of analytical grade and were used as received without further purification. All water for the aqueous solution was purified using a water purification system (resistivity, 18.2 MΩ·cm at 25°C, Daihan Scientific, Republic of Korea).

Synthesis of Au nanoparticles

The procedure to synthesize Au nanoparticles with diameter of 13 nm, 25 nm, 65 nm, and 109 nm was followed by described elsewhere [1–3].

<u>I. Synthesis of Au nanoparticles with a diameter of 13 nm.</u>

Inject 47.5 mL of deionized water into a 100 mL two-neck round bottom flask and heat it to 90°C. Prepare the pre-mixing solution by adding 0.5 mL of $HAuCl_4$ (1 wt%), 46.5 μL of $AgNO_3$ (0.1 wt%), 1.5 mL of citrate (1 wt%), and then adding deionized water to reach a total volume of 2.5 mL. Incubate the $HAuCl_4/AgNO_3$/citrate mixture solution for 5 min and rapidly add it to the boiling water under vigorously stirring. The reaction completes within 20 min, and once the clear solution turns red, remove the heat source and allow it to cool to room temperature with gentle stirring.

<u>II. Synthesis of Au nanoparticles with a diameter of 25 nm.</u>

For the synthesis of 25 nm Au nanoparticles, all procedures were identical to the method used for 13 nm Au nanoparticles, except for the volumes of $AgNO_3$ and citrate. Inject 95 mL of deionized water into a 250 mL two-neck round bottom flask and heat it to 90°C. Prepare the pre-mixing solution by adding 1 mL of $HAuCl_4$ (1 wt%), 85 μL of $AgNO_3$ (0.1 wt%), and 1.4 mL of citrate (1 wt%), and then add deionized water to reach a total volume of 5 mL. Incubate the $HAuCl_4/AgNO_3$/citrate mixture solution for 5 min, and rapidly add it to the boiling water while vigorously stirring. The reaction is completed in approximately 20 min, and once the clear solution turns red, remove the heat source and allow it to cool to room temperature with gentle stirring.

<u>III. Synthesis of Au nanoparticles with a diameter of 65 nm.</u>

In the first step, quickly inject freshly prepared 0.6 mL of $NaBH_4$ (10 mM) into a 10 mL mixture solution containing CTAB (100 mM) and $HAuCl_4$ (0.25 mM). Mix the solution for 2 min at 300 rpm and place it in a 27°C water bath for 3 h to remove any unreacted $NaBH_4$. In the second step, 4 mL of CTAC (200 mM), 3 mL of AA (100 mM), and 0.1 mL of the CTAB-capped Au cluster obtained in the previous step were placed in a 20 mL glass vial. Rapidly inject 4 mL of $HAuCl_4$ (0.5 mM) into the mixture solution and allow it to react in a 27°C water bath for 15 min. After the reaction is complete, the resulting solution was centrifuged at 12000 rpm for 30 min to remove any unreacted reagents. For the third step, mix 20 mL of CTAC (100 mM), 1.3 mL of AA (10 mM), and 0.1 mL of the 10 nm seed obtained in the second step were placed in a 100 mL flask. Inject 20 mL of $HAuCl_4$ (0.5 mM) into the mixture solution at a rate of 20 mL/h using syringe pump. Allow the reaction to proceed in a 27°C water bath for 10 min. Finally, centrifuge the final product at 12000 rpm for 10 min to wash and remove any remaining reactants.

<u>IV. Synthesis of Au nanoparticles with a diameter of 109 nm.</u>

The detailed preparation method of the Au cluster as a first seed is identical to the procedure for 65 nm of Au nanoparticle. The solution is immediately used in the next step. In the second step, in a 100 mL flask under magnetic stirring (700 rpm) at room temperature, mix 20 mL of CTAC (100 mM) aqueous solution, 15 mL of AA (100 mM), 0.4 mL of the solution from step 1, and 20 mL of $HAuCl_4$ (0.5 mM). The mixture solution was maintained for 30 min with stirring. The resulting solution is then centrifuged at 12000 rpm for 30 min to remove any excess reagents. In the third step, prepare a 40 mL aqueous solution of CTAC (100 mM), 15 mL of AA (10 mM), and 30 μL of the solution from step 2 in a 100 mL flask. Use a syringe pump to inject 10 mL of $HAuCl_4$ (2 mM) at a rate of 10 mL/h. The final product is washed with centrifugation at 2850 rpm for 25 min.

Synthesis of Pd nanoparticles

12 nm Pd NPs was synthesized by the nucleation and growth method [4]. 50 nm Pd NPs and 108 nm Pd NPs were synthesized by ethanol reduction method [5].

I. Synthesis of Pd nanoparticles with a diameter of 12 nm.

400 mg of PVP was dissolved in 2 mL ethylene glycol in 20 mL glass vial (Vial 1). The vial 1 was place into a 160°C oil bath. 1 mL of Na_2PdCl_4 (0.544 mM) solution in ethylene glycol was quickly injected into vial 1 under gentle stirring. After 1 h, the product was cooled in an ice-water bath. After the reaction is complete, the solution was washed once in acetone and twice in deionized water to remove excess reagents.

II. Synthesis of Pd nanoparticles with a diameter of 50 nm and 108 nm.

0.0224 g palladium (II) acetate was placed into a 100 mL of a two-neck flask. By stirring at 800 rpm for 10 min, 13 mL of DMSO was added to obtain a homogeneous solution at room temperature. The flask was then placed in a 25°C water bath with vigorous stirring for preheating. Subsequently, 17 mL of ethanol was added to the solution. The temperature was raised to 70°C and maintained for 1 h. The product was then collected by centrifugation at 8000 rpm for 10 min, and the supernatants were gently removed from the conical tube. The final product was redispersed in 1 mL of ethanol. For the preparation of 108 nm Pd nanoparticles, all procedures were exactly the same as the method for 50 nm, except that the reaction time was increased to 2 h instead of 1 h.

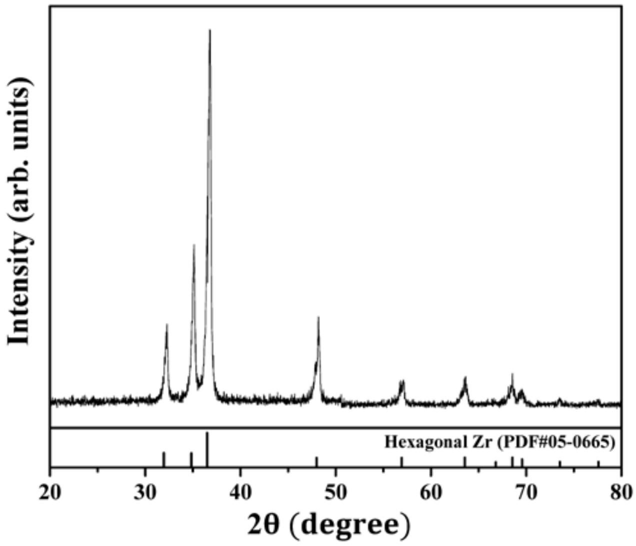

Figure S1. XRD spectrum of a zirconium pellet obtained before the corrosion test; hexagonal Zr diffraction pattern (PDF#05-0665) was obtained from PDF-2 database.

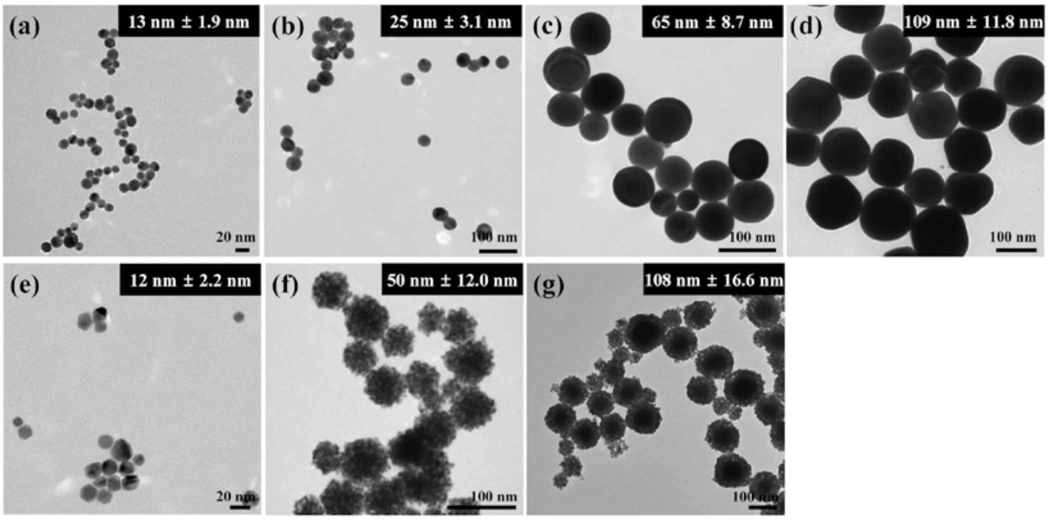

Figure S2. Representative TEM images of (**a**–**d**) Au and (**e**–**g**) Pd nanoparticles. TEM images in (**a** and **c**–**g**) are identical to those shown in Figure 3.

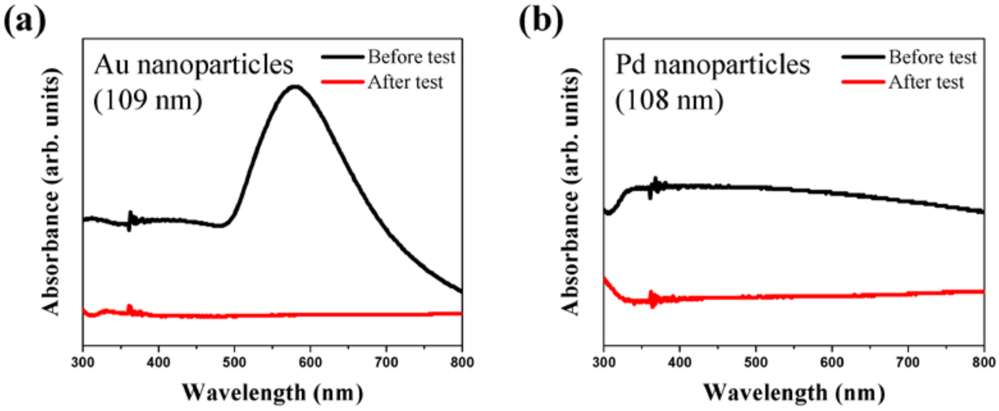

Figure S3. UV–vis absorption spectra for (**a**) 109 nm Au and (**b**) 108 nm Pd nanoparticles before (black line) and after (red line) the corrosion test.

References

1. Zheng, Y.; Zhong, X.; Li, Z.; Xia, Y. Successive, Seed-Mediated Growth for the Synthesis of Single-Crystal Gold Nanospheres with Uniform Diameters Controlled in the Range of 5–150 nm. *Part. Part. Syst. Charact.* **2014**, *31*, 266–273.
2. Xia, H.; Xiahou, Y.; Zhang, P.; Ding, W.; Wang, D. Revitalizing the Frens Method To Synthesize Uniform, Quasi-Spherical Gold Nanoparticles with Deliberately Regulated Sizes from 2 to 330 nm. *Langmuir* **2016**, *32*, 5870–5880.
3. Khlebtsov, B.N.; Tumskiy, R.S.; Burov, A.M.; Pylaev, T.E.; Khlebtsov, N.G. Quantifying the Numbers of Gold Nanoparticles in the Test Zone of Lateral Flow Immunoassay Strips. *ACS Appl. Nano Mater.* **2019**, *2*, 5020–5028.
4. Wang, Y.; Biby, A.; Xi, Z.; Liu, B.; Rao, Q.; Xia, X. One-Pot Synthesis of Single-Crystal Palladium Nanoparticles with Controllable Sizes for Applications in Catalysis and Biomedicine. *ACS Appl. Nano Mater.* **2019**, *2*, 4605–4612.
5. Wang, L.-M.; He, S.; Cui, Z.-M.; Guo, L. One-step synthesis of monodisperse palladium nanosphere and their catalytic activity for Suzuki coupling reactions. *Inorg. Chem. Commun.* **2011**, *14*, 1574–1578.
